# Efficacy of First-Line Nivolumab Plus Chemotherapy in Advanced Gastric Cancer Stratified by PD-L1 Expression: A Real-World Comparison

**DOI:** 10.3390/cancers17223716

**Published:** 2025-11-20

**Authors:** Dae-Ho Choi, Ji Eun Shin, Eunbyeol Lee, Seung Tae Kim, Sung Hee Lim

**Affiliations:** 1 Division of Hematology-Oncology, Department of Medicine, Samsung Medical Center, Sungkyunkwan University School of Medicine, Seoul 06351, Republic of Korea; daeho619.choi@samsung.com (D.-H.C.); jieun726.shin@samsung.com (J.E.S.); shty1@skku.edu (S.T.K.); 2 Biointelligence Center, Samsung Precision Genome Medicine Institute, Samsung Medical Center, Seoul 06351, Republic of Korea; ebyeol44.lee@sbri.co.kr

**Keywords:** gastric cancer, nivolumab, PD-L1, immunotherapy, biomarker

## Abstract

**Background:** Nivolumab plus chemotherapy has shown significant benefits in advanced gastric cancer (AGC) patients with PD-L1 combined positive score (CPS) 5 or higher. However, real-world data on its efficacy across different PD-L1 expression levels are limited. **Methods:** We conducted a retrospective analysis of 143 AGC patients treated with first-line nivolumab plus chemotherapy. Patients were stratified by PD-L1 CPS. Progression-free survival (PFS), overall survival (OS), and clinical factors affecting outcomes were evaluated. **Results:** Among 143 patients, 87 (60.8%) were classified as PD-L1 CPS < 5 and 56 (39.2%) as CPS ≥ 5. The PD-L1 ≥ 5 group had a higher proportion of dMMR and TMB-high. Notably, patients in the PD-L1 < 5 group also derived a PFS benefit from chemotherapy plus nivolumab, achieving a median PFS of 6.8 months, although outcomes were further enhanced in the PD-L1 ≥ 5 group with statistical significance (10.0 months; HR 0.56, *p* = 0.004). Although the PD-L1 ≥ 5 group showed a higher median OS compared with the PD-L1 < 5 group (26.2 vs. 18.8 months), the difference was not statistically significant (*p* = 0.234). Exploratory analyses demonstrated a stepwise trend toward improved survival with increasing PD-L1 cutoffs, with the most pronounced benefit observed in the PD-L1 ≥ 25 subgroup (PFS HR 0.28, *p* = 0.012; OS HR 0.21, *p* = 0.031). **Conclusions:** This real-world study suggests that nivolumab plus chemotherapy may benefit AGC patients across various PD-L1 expression levels, with trends favoring higher expression. These findings warrant further investigation in larger real-world studies to optimize patient selection and treatment strategies.

## Simple Summary

This study evaluated the real-world effectiveness of combining nivolumab with chemotherapy as first-line treatment in 143 patients with advanced HER2-negative gastric cancer. Outcomes were analyzed according to PD-L1 expression. As expected, patients with PD-L1 CPS ≥ 5 showed a significant benefit in progression-free survival compared with those with CPS < 5, while overall survival differences did not reach statistical significance. Importantly, even patients with CPS < 5 demonstrated clinically meaningful benefit, supporting the potential value of this regimen beyond the current CPS ≥ 5 threshold. Exploratory analyses showed a stepwise trend of greater efficacy with increasing PD-L1 expression, most evident in the CPS ≥ 25 subgroup. These findings highlight that nivolumab plus chemotherapy may offer benefit across PD-L1 expression levels and provide practical guidance for treating patients with low PD-L1 expression in real-world clinical settings.

## 1. Introduction

Gastric cancer is more common in East Asia than in the West and is the fourth most common cancer in South Korea [1,2]. Despite improvements in survival rates, advanced gastric cancer continues to have a poor prognosis, especially when surgery is not feasible [3,4]. The advent of immune checkpoint inhibitors (ICIs) has transformed the treatment landscape for advanced gastric cancer (AGC).

CheckMate-649 demonstrated that nivolumab plus chemotherapy resulted in significant improvements in overall survival (OS; 14.4 vs. 11.1 months; HR = 0.71; *p* < 0.0001) and progression-free survival (PFS; 7.7 vs. 6 months; HR = 0.68; *p* < 0.0001) compared to chemotherapy alone in patients with a PD-L1 CPS of ≥5. Additional results also showed some improvement in OS and PFS in patients with a PD-L1 CPS of ≥1 (OS = 14 vs. 11.3 months, HR = 0.77; PFS = 7.5 vs. 6.9, HR = 0.74) and in all randomly assigned patients (OS = 13.8 vs. 11.6 months, HR = 0.8; PFS = 7.7 vs. 6.9, HR = 0.77) [5,6]. Both KEYNOTE-859 and RATIONALE-305 demonstrated that the addition of ICIs (pembrolizumab in KEYNOTE-859; tislelizumab in RATIONALE-305) to chemotherapy provided a statistically significant and clinically meaningful improvement in OS, PFS, and objective response rate in patients with HER2-negative AGC. The benefit was observed regardless of PD-L1 expression, although a more pronounced effect was seen in patients with higher PD-L1 expression [7,8].

However, a critical gap exists between clinical trial populations and real-world patient demographics. While approximately 60% of patients in CheckMate-649 had PD-L1 CPS ≥ 5, real-world data suggest this proportion may be closer to 30% in stage IV gastric cancer patients [9]. Furthermore, subgroup analyses have indicated a potential lack of benefit from nivolumab addition in patients with low PD-L1 expression (CPS < 5) [10]. In South Korea, reimbursement for nivolumab plus chemotherapy is currently limited to patients with PD-L1 CPS ≥ 5, based on CheckMate-649 results.

Real-world data on the efficacy of this regimen in patients with lower PD-L1 expression are scarce. This retrospective study aims to address this knowledge gap by investigating the efficacy of nivolumab plus chemotherapy in advanced gastric cancer patients stratified by PD-L1 CPS levels, comparing outcomes between those with CPS < 5 and ≥5.

## 2. Materials and Methods

### 2.1. Patient Selection and Data Collection

We investigated patients diagnosed with recurrent or metastatic HER2-negative gastric cancer at Samsung Medical Center between January 2021 and December 2023 who received first-line cytotoxic chemotherapy (XELOX or FOLFOX) in combination with nivolumab. We reviewed electronic medical records (EMR) and extracted data on age, sex, Eastern Cooperative Oncology Group (ECOG) performance status, pathology, Epstein–Barr virus (EBV) status, programmed death-ligand 1 (PD-L1) expression, microsatellite instability (MSI), tumor mutational burden (TMB), and the presence and location of distant metastases at treatment initiation.

This study was approved by the Institutional Review Board (IRB) of Samsung Medical Center with the requirement for informed consent waived (IRB No. 2024-03-061).

### 2.2. Biomarker and Genomic Analysis

EBV status was assessed using in situ hybridization (ISH). PD-L1 expression was evaluated using either the 22C3 pharmDx assay (Agilent Technologies, Santa Clara, CA, USA) or the 28-8 pharmDx assay (Agilent Technologies) and reported as combined positive score (CPS).

For genomic analysis, tumor regions were micro-dissected from formalin-fixed paraffin-embedded (FFPE) tissue, and genomic DNA was extracted using the AllPrep DNA/RNA FFPE Kit (Qiagen, Hilden, Germany). DNA concentration was measured with the Qubit dsDNA HS Assay Kit (Thermo Fisher Scientific, Waltham, MA, USA), and 40 ng of DNA was used for library preparation. DNA integrity was assessed using the Genomic DNA ScreenTape assay on an Agilent 2200 TapeStation System. DNA libraries were prepared using two targeted NGS assays: the TruSight Oncology 500 (TSO 500) assay (Illumina, San Diego, CA, USA) and the Oncomine Comprehensive Assay Plus (OCA Plus) (Thermo Fisher Scientific, Waltham, MA, USA), following the manufacturers’ protocols.

For TSO 500, hybrid capture-based enrichment was applied to DNA and RNA from FFPE samples, incorporating unique molecular identifiers (UMIs) to reduce sequencing and deamination artifacts. Variants including single nucleotide variants (SNVs), small insertions/deletions (indels), copy number variations (CNVs), and gene fusions were identified using the TSO 500 pipeline (Local App version 1.3.0.39). Variants were annotated with the Ensembl Variant Effect Predictor (VEP) against public databases (dbSNP, gnomAD, ClinVar, COSMIC, RefSeq, Ensembl) and classified according to ASCO/CAP recommendations. TMB and MSI were also derived from the TSO 500 pipeline.

For OCA Plus, multiplex PCR-based amplification targeted hotspot mutations, CNVs, and fusions across a broader set of clinically relevant cancer genes compared to earlier Oncomine panels. Sequencing was performed on the Ion Torrent platform (Thermo Fisher Scientific), and data were analyzed through the manufacturer’s integrated pipeline to detect SNVs, indels, CNVs, and gene fusions. Variant annotation and classification were performed with reference to public databases and established clinical guidelines, ensuring consistency with TSO 500 analysis.

### 2.3. Outcomes

The primary objective was to compare PFS of first line nivolumab plus chemotherapy between patients with PD-L1 CPS levels of less than 5 and patients with PD-L1 CPS levels of 5 or more. PFS is defined as the time from initiation of nivolumab plus chemotherapy to disease progression or any cause of death. OS is defined as the time from the date of initiation of nivolumab plus chemotherapy to the date of death from any cause.

### 2.4. Statistical Analysis

Patients were divided into two groups for analysis: PD-L1 CPS < 5 and PD-L1 CPS ≥ 5. Chi-squared test was used to determine the differences between the groups. Characteristics of each group were expressed as numbers and frequencies as percentages. PFS and OS were analyzed using the Kaplan–Meier method with 95% confidence intervals. The log-rank test was used for comparisons between the two groups. Two-sided tests were performed for all *p*-values, and statistical significance was defined as *p* < 0.05. All statistical analyses were performed with R version 4.2.2.

## 3. Results

### 3.1. Characteristics of the Study Population

Of the total 143 patients, 87 patients (60.8%) had PD-L1 CPS < 5 (PD-L1 < 5 group), and 56 patients (39.2%) had PD-L1 CPS 5 or higher (PD-L1 ≥ 5 group). The median age in both groups was 58 years. ECOG performance status was 0–1 for the majority of patients in both groups. The most common primary tumor location at initial diagnosis was the body (58.7%), followed by the antrum (25.9%), and the fundus (2.8%). Previous definitive surgery was performed in 29 (33.3%) patients in the PD-L1 < 5 group and 9 (16.1%) in the PD-L1 ≥ 5 group. The most common metastatic site was the peritoneum (56.6%), followed by the liver (18.9%), bone (7.7%), and lung (7.0%). Notably, liver metastasis was more frequent in the PD-L1 CPS ≥ 5 group than in the PD-L1 CPS < 5 group (12.5% vs. 28.6%, *p* = 0.03). Signet ring cell carcinoma was present in 17 (19.5%) patients in the PD-L1 CPS < 5 group and 4 (7.1%) in the PD-L1 CPS ≥ 5 group. EBV ISH positivity was observed in 3 patients (2.1%). MLH1 loss was observed in two (3.1%) patients in the PD-L1 CPS < 5 group and nine (18.4%) in the PD-L1 CPS ≥ 5 group (*p* = 0.02). Tumor mutation burden (TMB) differed significantly between the two groups, with a higher frequency of TMB-high observed in the PD-L1 CPS ≥ 5 group (*p* = 0.008). In mutation analysis, KRAS mutations were more frequent in the PD-L1 CPS ≥ 5 group (*p* = 0.036), whereas PIK3CA, TP53, and ARID1A mutations showed no significant differences. Nivolumab plus XELOX regimen was more commonly used in both groups (Table 1).

### 3.2. Survival Outcomes According to PD-L1 Expression Status

The data cutoff date was 30 June 2025. At the time of data cutoff, the median duration of follow-up was 33.4 months (95% CI, 28.9–35.7). In the overall study population, the median progression-free survival (PFS) was 8.8 months (95% CI, 6.7–10.7), and the median overall survival (OS) was 19.0 months (95% CI, 17.1–26.8).

**Figure 1:**
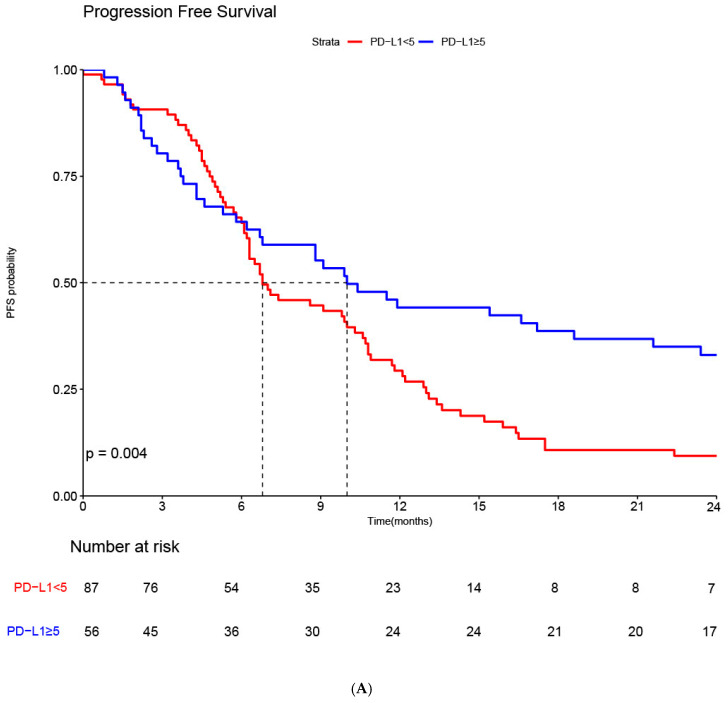

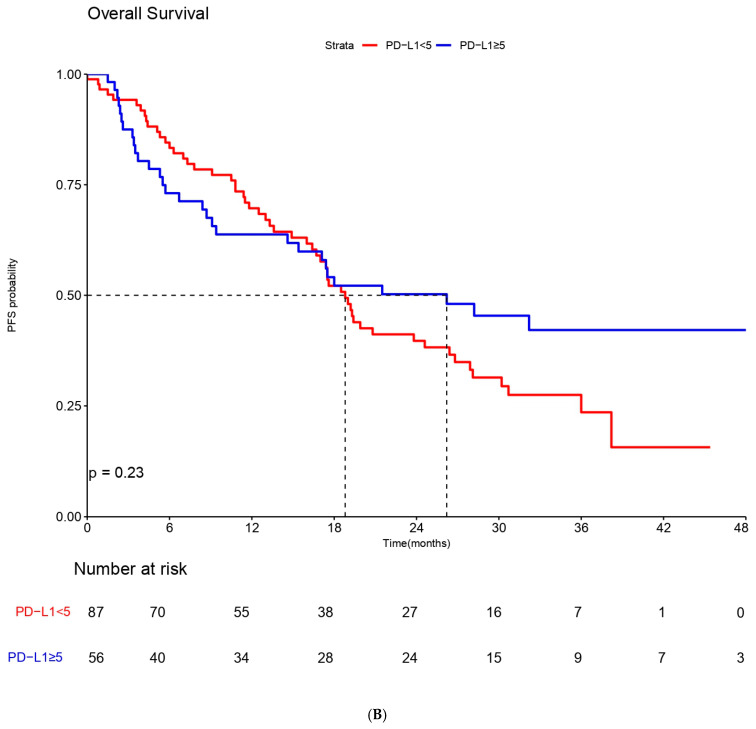
(**A**) Kaplan–Meier analysis of Progression-Free Survival (PFS) stratified by Programmed Death-Ligand 1 (PD-L1) Combined Positive Score (CPS). A total of 75 progression events were observed in the PD-L1 CPS < 5 group (N = 87) and 37 progression events were observed in the PD-L1 CPS ≥ 5 group (N = 56). Survival curves were compared using the log-rank test. (**B**) Kaplan–Meier analysis of Overall Survival (OS) stratified by Programmed Death-Ligand 1 (PD-L1) Combined Positive Score (CPS). A total of 56 death events were observed in the PD-L1 CPS < 5 group (N = 87) and 30 death events were observed in the PD-L1 CPS ≥ 5 group (N = 56). Survival curves were compared using the log-rank test.

When stratified by PD-L1 expression status, patients with PD-L1 < 5 had a median PFS of 6.8 months (95% CI, 6.3–10.6), whereas those with PD-L1 ≥ 5 achieved a median PFS of 10.0 months (95% CI, 6.7–23.4), demonstrating a statistically significant improvement in PFS compared with the PD-L1 < 5 group (*p* = 0.004) (Figure 1A). For OS, patients with PD-L1 < 5 had a median of 18.8 months (95% CI, 16.7–26.4), while those with PD-L1 ≥ 5 had a median OS of 26.2 months (95% CI, 15.4–not reached). Although a numerical improvement was observed in the PD-L1 ≥ 5 group, the difference did not reach statistical significance (*p* = 0.23) (Figure 1B).

**Figure 2:**
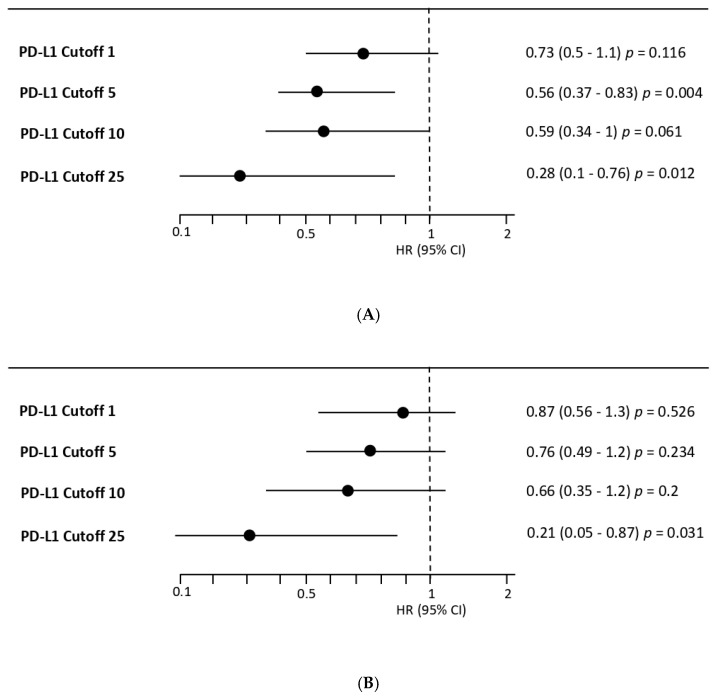
(**A**) Forest plot of Hazard Ratios (HRs) for Progression-Free Survival (PFS) according to different Programmed Death-Ligand 1 (PD-L1) expression cutoffs. HRs and 95% Confidence Intervals (CIs) were derived from a Cox proportional hazards model. An HR < 1 favors the nivolumab plus chemotherapy arm. The vertical dashed line indicates an HR of 1.0. (**B**) Forest plot of Hazard Ratios (HRs) for Overall Survival (OS) according to different Programmed Death-Ligand 1 (PD-L1) expression cutoffs. HRs and 95% Confidence Intervals (CIs) were derived from a Cox proportional hazards model. An HR < 1 favors the nivolumab plus chemotherapy arm. The vertical dashed line indicates an HR of 1.0.

Exploratory analyses were performed to further assess the impact of PD-L1 expression at different cutoff thresholds (CPS 1, 5, 10, and 25) (Figure 2A,B). Using CPS 1 as the cutoff, patients with PD-L1 ≥ 1 showed a modest improvement in PFS (median, 8.8 vs. 7.4 months; HR, 0.73; 95% CI, 0.50–1.10; *p* = 0.116) and OS (median, 19.9 vs. 19.0 months; HR, 0.87; 95% CI, 0.56–1.30; *p* = 0.526), although the differences were not statistically significant. When applying a CPS 10 threshold, the PD-L1 ≥ 10 group demonstrated a longer PFS compared with the PD-L1 < 10 group (median, 10.2 vs. 7.1 months; HR, 0.59; 95% CI, 0.34–1.00; *p* = 0.061), with a trend toward improved OS (median not reached vs. 18.8 months; HR, 0.66; 95% CI, 0.35–1.20; *p* = 0.200), although these results did not reach statistical significance. Notably, with CPS 25 as the cutoff, the benefit became more pronounced: the PD-L1 ≥ 25 subgroup exhibited significantly prolonged PFS (median not reached vs. 7.1 months; HR, 0.28; 95% CI, 0.10–0.76; *p* = 0.012) and OS (median not reached vs. 18.5 months; HR, 0.21; 95% CI, 0.05–0.87; *p* = 0.031), suggesting that higher PD-L1 expression levels may be associated with greater survival benefit with combination treatment of nivolumab. There appeared to be a stepwise trend in survival outcomes, with higher PD-L1 cutoff levels corresponding to progressively greater improvements in both PFS and OS.

### 3.3. Subgroup for PFS

**Figure 3:**
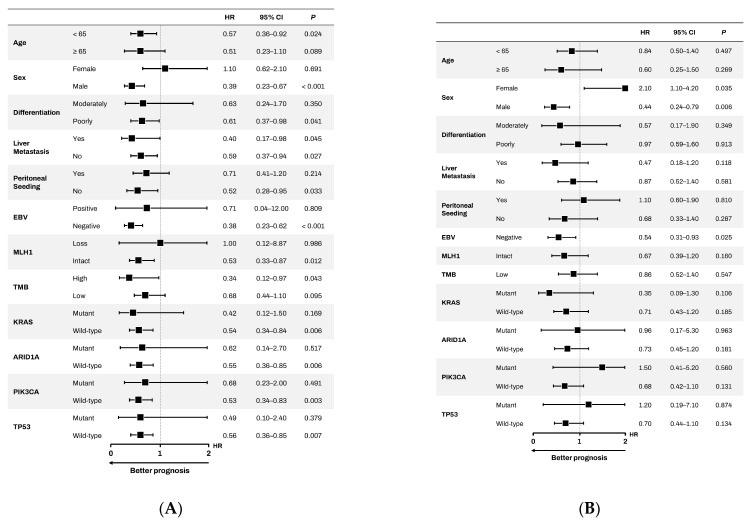
(**A**) Subgroup analysis of Hazard Ratios (HRs) for Progression-Free Survival (PFS). HRs and 95% Confidence Intervals (CIs) were calculated using a Cox proportional hazards model for each prespecified subgroup. An HR < 1 favors the nivolumab plus chemotherapy arm. *p*-values for interaction were calculated to assess the consistency of the treatment effect across subgroups. (**B**) Subgroup analysis of Hazard Ratios (HRs) for Overall Survival (OS). HRs and 95% Confidence Intervals (CIs) were calculated using a Cox proportional hazards model for each prespecified subgroup. An HR < 1 favors the nivolumab plus chemotherapy arm. *p*-values for interaction were calculated to assess the consistency of the treatment effect across subgroups.

We conducted subgroup analyses based on baseline characteristics. The superior efficacy of chemotherapy plus nivolumab in patients with PD-L1 CPS ≥ 5 was particularly pronounced and statistically significant across several subgroups (Figure 3A). Notably, male patients experienced a substantial PFS benefit (HR = 0.39, *p* < 0.001), a finding not mirrored in their female counterparts (HR = 1.1, *p* = 0.691). A significant advantage was also observed in patients younger than 65 years (HR = 0.57, *p* = 0.024) and in those whose primary tumor was located in the body of the stomach (HR = 0.43, *p* = 0.005). Furthermore, patients without liver metastasis demonstrated a significantly improved PFS (HR = 0.59, *p* = 0.027), and a borderline significant benefit was also seen in patients with liver metastasis (HR = 0.4, *p* = 0.045). There was no substantial difference between the two groups with respect to genomic alterations, dMMR, or TMB status. However, the number of patients harboring genomic alterations or classified as dMMR or TMB-high was too small to allow for meaningful statistical comparison or subgroup analysis.

In contrast to the clear benefits observed in PFS, the analysis of OS did not yield statistically significant advantages for any of the examined subgroups (Figure 3B). While some trends toward improved OS were noted, such as in male patients (HR = 0.7, *p* = 0.181), without statistical significance. The significant PFS advantages seen in subgroups defined by age, tumor location, and presence of liver metastasis did not translate into a corresponding significant OS benefit.

### 3.4. Genomic Landscape by PD-L1 Expression Status

**Figure 4:**
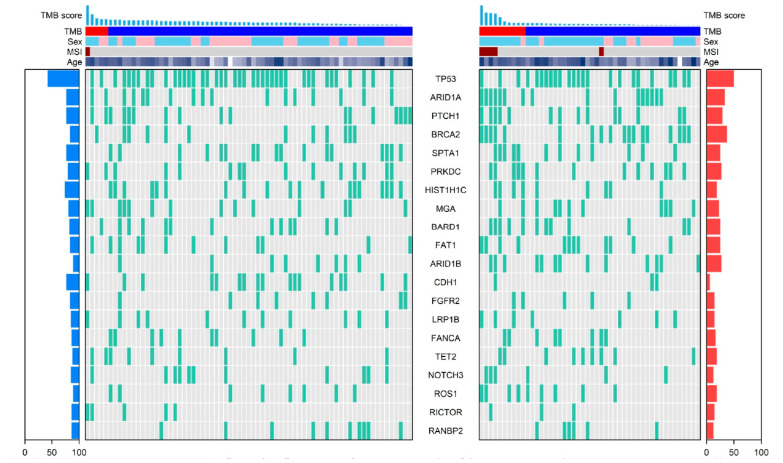
Heatmap of Genomic Alterations Stratified by PD-L1 expression (Left: PD-L1 CPS < 5; Right: PD-L1 CPS ≥ 5). Color annotations are as follows: in the TMB bar, red indicates TMB-High (≥10 mut/Mb) and blue indicates TMB-Low (<10 mut/Mb). In the sex annotation, light blue denotes male and pink denotes female. MSI status is shown as brown for MSI-H and gray for MSS or MSI-L. Age is represented by a continuous color scale, with darker shading indicating older age.

Next-generation sequencing (NGS) data were available for 119 patients with sufficient tissue (71 in the PD-L1 < 5 group and 48 in the PD-L1 ≥ 5 group). Figure 4 displays a heatmap of the genomic landscape, allowing for a comparison of the two cohorts. The “genomic alterations” plotted represent pathogenic somatic mutations (Single Nucleotide Variants, SNVs, and indels) and Copy Number Variations (CNVs) in key cancer-related genes, as detailed in our Section 4.

Several core driver mutations were highly prevalent across both groups, consistent with the known molecular biology of gastric cancer. Pathogenic mutations in TP53 and ARID1A were the most frequently observed alterations and were distributed comparably across both the PD-L1 < 5 and ≥5 groups, suggesting these alterations are fundamental oncogenic drivers largely independent of the tumor’s immune phenotype. Other frequently altered genes in both groups included CDH1, BRCA2, and FGFR2.

Figure 4 also illustrates that PD-L1 expression correlates with other established immunotherapy response biomarkers. As shown in the heatmap annotation, both TMB-high (TMB-H) and MSI-high (MSI-H) statuses were markedly enriched in the PD-L1 ≥ 5 group. EBV-positive tumors, another immunogenic subset, were also predominantly found in the PD-L1 ≥ 5 cohort. Collectively, these results indicate that while PD-L1-low tumors share common driver mutations with PD-L1-high tumors, the latter display a distinct immunogenic genomic profile.

## 4. Discussion

In this retrospective cohort of patients with recurrent or metastatic HER2-negative gastric cancer receiving first-line nivolumab plus chemotherapy, we found that PD-L1 expression was associated with different clinical outcomes. Although patients with PD-L1 CPS < 5 also derived a clinically meaningful benefit, the improvement was more pronounced and reached statistical significance in the CPS ≥ 5 subgroup (median PFS, 10.0 vs. 6.8 months; HR 0.56; *p* = 0.004), whereas OS showed only a numerical advantage without statistical significance (26.2 vs. 18.8 months; HR 0.76; *p* = 0.234). Exploratory analyses across multiple thresholds suggested a stepwise gradient of benefit, with increasing PD-L1 cutoffs corresponding to progressively greater improvements in both PFS and OS, most evident in the CPS ≥ 25 subgroup.

The advent of ICIs combined with platinum-based chemotherapy has revolutionized first-line treatment for HER2-negative advanced gastric cancer. However, there remains a lack of consensus regarding the use of ICI combinations in patients with low PD-L1 expression. Previous studies have suggested that PD-L1 expression can be a robust predictor of OS benefit from ICI in gastric cancer [11,12,13]. Subgroup analysis from pivotal trials such as CheckMate-649 and KEYNOTE-859 indicated that first-line ICI plus chemotherapy is less effective in patients with low PD-L1 expression. In CheckMate-649, the primary endpoint focused on CPS ≥ 5, where nivolumab plus chemotherapy showed a clear OS benefit (14.4 vs. 11.1 months; HR 0.71). By contrast, in the CPS < 5 subgroup, the advantage was minimal (OS; 13.1 vs. 12.9 months; HR ~0.94), indicating little survival benefit. In KEYNOTE-859, the trial demonstrated a significant OS improvement in the overall ITT population (12.9 vs. 11.5 months; HR 0.78; not significant). Importantly, even in CPS < 5 patients, pembrolizumab plus chemotherapy yielded a modest but consistent benefit (median OS 11.5 vs. 11.1 months; HR 0.78; *p* = 0.013), suggesting broader efficacy irrespective of PD-L1 status [7,8]. Consistent findings were also reported in other global and regional trials such as ATTRACTION-4 [14,15], KEYNOTE-590 (esophagogastric cohort) [16], and ORIENT-16 [17], where sintilimab plus chemotherapy significantly improved OS (HR 0.77) in a Chinese population.

Our findings suggest that patients with PD-L1 CPS < 5 may still derive meaningful clinical benefit from nivolumab addition to chemotherapy. While the magnitude of benefit was less pronounced compared to higher PD-L1 expression groups, the median PFS of 6.8 months in the PD-L1 < 5 cohort demonstrates clinically relevant outcomes. This observation aligns with emerging evidence from pivotal clinical trials, where ICIs plus chemotherapy showed consistent benefit even in low PD-L1 expression patients, suggesting that immunotherapy efficacy may not be entirely dependent on PD-L1 expression levels in gastric cancer.

Establishing an optimal threshold for low PD-L1 expression remains challenging [18]. Issues persist regarding the interpretation of PD-L1 expression [19]. While early study suggested some evidence for the potential interchangeability between the 22C3 pharmDx and 28-8 pharmDx assays in gastric cancer [20], subsequent reports highlighted suboptimal concordance rates of PD-L1 CPS by 28-8 and 22C3 assay [21]. Patients with discordant results between these assays showed poor PFS when treated with nivolumab plus chemotherapy compared to those with concordantly positive PD-L1 expression [22]. A recent meta-analysis indicated that the optimal threshold where patients significantly benefit from adding ICIs therapy is PD-L1 CPS ≥ 5 [23], however there are insufficient data to identify an ideal CPS cut point through meta-analysis alone.

In this study, we observed significant differences in baseline characteristics between PD-L1 CPS ≥ 5 and <5 groups. Liver metastasis was also more frequent in the PD-L1 CPS ≥ 5 group. Real-world data also showed that the higher prevalence of dMMR tumors in the PD-L1 CPS ≥ 5 group (18.4%) compared to the PD-L1 CPS < 5 group (3.1%). This observation is consistent with previous reports that dMMR or microsatellite instability-high (MSI-H) gastric cancers typically exhibit high PD-L1 expression [24,25].

The NGS results revealed a higher frequency of PIK3CA and KRAS mutations in the PD-L1 CPS ≥ 5 group compared to the PD-L1 CPS < 5 group. This finding aligns with previous studies demonstrating increased PD-L1 expression in patients with KRAS mutations. The RAS pathway has been shown to regulate PD-L1 expression as a mechanism for tumor immune evasion [26,27]. Similarly, PIK3CA mutations correlate with PD-L1 expression, given their shared involvement in the RTK pathway [28]. These findings suggest the potential for combining ICIs with KRAS or PIK3CA targeted therapies, warranting further investigation.

The superior efficacy of chemotherapy plus nivolumab in patients with PD-L1 CPS ≥ 5 was pronounced in particular subgroups. Male patients showed greater improvement, consistent with reported sex-based differences in immune responses [29]. Patients with primary tumors in the gastric body and those without liver metastasis also derived more benefit, suggesting that tumor location and metastatic status influence the tumor microenvironment and response to immunotherapy [30]. Nevertheless, no study has specifically evaluated the impact of primary tumor location on ICI effectiveness in gastric cancer. These findings highlight that, in addition to PD-L1 expression, clinical and biological factors modulate immunotherapy efficacy and should be integrated into personalized treatment strategies.

The discordance between PFS and OS outcomes warrants further discussion. While nivolumab plus chemotherapy demonstrated significant PFS benefit in patients with PD-L1 CPS ≥ 5 compared to those with CPS < 5 (10.0 vs. 6.8 months; HR 0.56, *p* = 0.004), this advantage did not translate into statistically significant OS differences (26.2 vs. 18.8 months; HR 0.76, *p* = 0.234). This pattern suggests that subsequent therapeutic interventions may have attenuated the initial first-line treatment differences. In our cohort, patients in both PD-L1 groups received various second line and beyond treatments, which may have contributed to the convergence of OS outcomes despite the initial PFS differences.

Importantly, our real-world findings are consistent with those of international RWE studies such as G-KNIGHT and TOG, further supporting the durability of chemoimmunotherapy benefit beyond clinical trial settings. In both studies, nivolumab plus chemotherapy achieved median OS outcomes comparable to or exceeding those reported in pivotal trials, reinforcing the clinical applicability of ICI-based regimens in diverse real-world populations [31,32]. Notably, our real-world cohort demonstrated prolonged OS outcomes compared to historical pivotal trials, particularly in patients with PD-L1 CPS < 5. While CheckMate-649 reported minimal OS benefit in the CPS < 5 subgroup (median OS 13.1 vs. 12.9 months), our PD-L1 < 5 patients achieved a median OS of 18.8 months, suggesting improved outcomes in routine clinical practice. This enhancement may be attributed to several factors including refined patient selection, optimized supportive care, and potentially different baseline characteristics in our Asian population compared to the global trial cohorts.

The question of whether patients with PD-L1 < 5 can benefit from ICIs remains critical and directly linked to how we refine patient selection. In our PD-L1–low cohort, we identified subgroups with EBV positivity, dMMR, or high TMB, all of which are recognized as markers of immunotherapy sensitivity [33,34,35,36]. Notably, Sialic acid-binding immunoglobulin-like lectin 15 (SIGLEC-15) has emerged as a novel immune checkpoint that may drive immune evasion in PD-L1-low tumors [31]. Studies in gastric cancer show SIGLEC-15 expression is often inversely correlated with PD-L1, suggesting it acts as a compensatory suppressive mechanism. These biological mechanisms collectively support the concept that PD-L1–independent immune activation pathways can contribute to ICI responsiveness, as recently reviewed in a comprehensive mechanistic study on nivolumab- and ipilimumab-based immunotherapy [37]. These findings suggest that even PD-L1–low patients may benefit from ICI therapy, underscoring the need for broader biomarker research (including dMMR, EBV, and SIGLEC-15) to optimize treatment.

Our study has limitations. Single-center retrospective design with a limited sample size, which may have contributed to the lack of statistically significant prognostic differences despite observed trends. Differences in baseline characteristics between groups could potentially bias prognostic comparisons; however, these differences may be inherently related to PD-L1 expression levels rather than introducing true prognostic bias. In addition, the inclusion of patients who underwent salvage surgery may have influenced overall survival outcomes.

## 5. Conclusions

In conclusion, our real-world cohort demonstrated that ICI combined with chemotherapy provided clinical benefit even in patients with PD-L1 CPS < 5, although consistent with prior studies, the magnitude of benefit was more pronounced in those with higher PD-L1 expression. These findings not only confirm the clinical utility of ICIs across PD-L1 subgroups in routine practice but also provide valuable insight into the use of ICIs in PD-L1–low populations. Further large-scale, real-world data are needed to validate these results and explore the subgroup patients with potential benefit from combining immunotherapy with chemotherapy in the low PD-L1 expression gastric cancer.

## Figures and Tables

**Table 1:** Baseline characteristics.

Characteristics	PD-L1 < 5 (*n* = 87)	PD-L1 ≥ 5 (*n* = 56)	*p*-Value
Median age (range)	58 (24–76)	58 (21–79)	0.22
Age < 65 years	71 (81.6%)	40 (71.4%)	
Age ≥ 65 years	16 (18.4%)	16 (28.6%)	
Sex			0.08
Men	45 (51.7%)	38 (67.9%)	
Women	42 (48.3%)	18 (32.1%)	
ECOG performance status			0.70
0–1	86 (98.9%)	54 (96.4%)	
≥2	1 (1.1%)	2 (3.6%)	
Primary tumor location at initial diagnosis			0.03
Cardia	12 (13.8%)	6 (10.7%)	
Fundus	2 (2.3%)	2 (3.6%)	
Body	58 (66.7%)	26 (46.4%)	
Antrum	15 (17.2%)	22 (39.3%)	
Previous surgery (for definitive aim)			0.03
Yes	29 (33.3%)	9 (16.1%)	
No	58 (67.7%)	47 (83.9%)	
Site of metastases			
Liver	11 (12.5%)	16 (28.6%)	0.03
Peritoneum	55 (63.2%)	26 (46.4%)	0.07
Lung	8 (9.1%)	2 (3.6%)	0.34
Bone	10 (11.4%)	1 (1.8%)	0.07
Signet ring cell carcinoma			0.07
Yes	17 (19.5%)	4 (7.1%)	
No	70 (80.5%)	52 (92.9%)	
EBV ISH			0.69
Positive	1 (1.4%)	2 (4.7%)	
Negative	68 (98.6%)	41 (95.3%)	
Unknown	18	13	
MLH1 IHC			0.02
Intact	62 (96.9%)	40 (81.6%)	
Loss	2 (3.1%)	9 (18.4%)	
Unknown	23	7	
Tumor mutation burden			0.008
Low (<10 mut/Mb)	75 (90.4%)	39 (70.9%)	
High (≥10 mut/Mb)	8 (9.6%)	16 (29.1%)	
Unknown	4	1	
Co-mutation confirmed by NGS			
PIK3CA	9 (10.6%)	10 (18.2%)	0.430
KRAS	5 (5.9%)	11 (20.0%)	0.036
TP53	7 (8.2%)	4 (7.3%)	0.958
ARID1A	7 (8.2%)	3 (5.5%)	0.805
No tier I/II mutation	42 (49.4%)	19 (34.5%)	
Chemotherapy regimens			0.30
† FOLFOX	13 (14.9%)	13 (23.2%)	
‡ XELOX	74 (85.1%)	43 (76.8%)	

PD-L1, Programmed cell death-ligand 1; ECOG, Eastern Cooperative Oncology Group; IHC, Immunohistochemistry; ISH, In situ hybridization; EBV, Epstein–Barr virus; MLH1, MutL protein homolog 1; NGS, Next-generation sequencing; PIK3CA, phosphatidylinositol-4,5-bisphosphate 3-kinase catalytic subunit alpha; KRAS, Kirsten rat sarcoma virus; TP53, tumor protein p53; ARID1A, AT-rich interaction domain 1A; † FOLFOX is fluorouracil plus oxaliplatin plus leucovorin. ‡ XELOX is capecitabine plus oxaliplatin.

## Data Availability

The data presented in this study are available on reasonable request from the corresponding author. The data are not publicly available due to privacy and ethical restrictions related to patient confidentiality and institutional policy at Samsung Medical Center.

## Abbreviations

The following abbreviations are used in this manuscript:

AGC	Advanced Gastric Cancer
CPS	Combined Positive Score
EBV	Epstein–Barr Virus
EMR	Electronic Medical Records
ECOG	Eastern Cooperative Oncology Group
ICI	Immune Checkpoint Inhibitor
ISH	In Situ Hybridization
MSI	Microsatellite Instability
MSI-H	Microsatellite Instability-High
NGS	Next-Generation Sequencing
OS	Overall Survival
PFS	Progression-Free Survival
TMB	Tumor Mutational Burden
